# Fluticasone furoate: once-daily evening treatment versus twice-daily treatment in moderate asthma

**DOI:** 10.1186/1465-9921-12-160

**Published:** 2011-12-21

**Authors:** Ashley Woodcock, Eugene R Bleecker, William W Busse, Jan Lötvall, Neil G Snowise, Lucy Frith, Loretta Jacques, Brett Haumann, Eric D Bateman

**Affiliations:** 1 School of Translational Medicine, University of Manchester, Manchester, UK; 2 Center for Genomics and Personalized Medicine, Wake Forest University Health Sciences, Winston-Salem, NC, USA; 3 Department of Medicine, University of Wisconsin, Madison, USA; 4 Krefting Research Centre, University of Gothenburg, Gothenburg, Sweden; 5 Respiratory Medicines Development Centre, GlaxoSmithKline, Uxbridge, UK; 6 Department of Medicine, University of Cape Town, Cape Town, South Africa

**Keywords:** Asthma, fluticasone furoate, inhaled corticosteroid, once daily, efficacy, safety

## Abstract

**Background:**

Inhaled corticosteroids are the recommended first-line treatment for asthma but adherence to therapy is suboptimal. The objectives of this study were to compare the efficacy and safety of once-daily (OD) evening and twice-daily (BD) regimens of the novel inhaled corticosteroid fluticasone furoate (FF) in asthma patients.

**Methods:**

Patients with moderate asthma (age ≥ 12 years; pre-bronchodilator forced expiratory volume in 1 second (FEV_1_) 40-85% predicted; FEV_1 _reversibility of ≥ 12% and ≥ 200 ml) were randomized to FF or fluticasone propionate (FP) regimens in a double-blind, crossover study. Patients were not permitted to have used any ICS for ≥ 8 weeks prior to enrolment and subsequently received doses of FF or FP 200 μg OD, FF or FP 100 μg BD and matching placebo by inhalation for 28 days each. Primary endpoint was Day 28 evening pre-dose (trough) FEV_1_; non-inferiority of FF 200 μg OD and FF 100 μg BD was assessed, as was superiority of all active treatment relative to placebo. Adverse events (AEs) and 24-hour urinary cortisol excretion were assessed.

**Results:**

The intent-to-treat population comprised 147 (FF) and 43 (FP) patients. On Day 28, pre-dose FEV_1 _showed FF 200 μg OD to be non-inferior (pre-defined limit -110 ml) to FF 100 μg BD (mean treatment difference 11 ml; 95% CI: -35 to +56 ml); all FF and FP regimens were significantly superior to placebo (p ≤ 0.02). AEs were similar to placebo; no serious AEs were reported. Urinary cortisol excretion at Day 28 for FF was lower than placebo (ratios: 200 μg OD, 0.75; 100 μg BD, 0.84; p ≤ 0.02).

**Conclusions:**

FF 200 μg OD in the evening is an efficacious and well tolerated treatment for asthma patients and is not inferior to the same total BD dose.

**Trial registration:**

Clinicaltrials.gov; NCT00766090.

## Background

A variety of treatments are available for asthma but there remains potential to improve the level of disease control in adults and children [1-4]. Failure to achieve asthma control affects patients' daily lives, for example through persistent symptoms, more frequent exacerbations and missed work and school time, placing demands on emergency care facilities [2,5]. Further improvements to the range of therapeutic options for asthma are needed so that patients can achieve better disease control.

Inhaled corticosteroids (ICS) are the most effective controller medications for the first-line treatment of asthma in adults and children and are also used at later stages in combination with other medications, specifically long-acting beta_2 _agonists (LABA) [6]. ICS are typically developed for twice-daily dosing but once-daily evening dosing of an ICS has been reported to significantly improve adherence to therapy compared with twice-daily dosing (93.3% vs. 89.3% [p < 0.001] as measured by automatic dose counter) in an open-label 12 week study of mometasone furoate [7]. This is a benefit that has the potential to improve patient outcomes, given the association between poor adherence rates (particularly for controller medications) and uncontrolled asthma in children and adults [8,9], and the reported correlation between falling rates of adherence to ICS and higher rates of asthma-related hospitalization in adults [10].

Animal and human pharmacology studies show that the novel ICS fluticasone furoate (FF) has a long duration of action and prolonged retention in the lung, suggesting that it is suitable for once-daily dosing [11]. FF and fluticasone propionate (FP) are structurally different. At the C-17α position, FF contains an ester derived from 2-furoic acid which replaces the simpler propionate ester. These differences mean that FF has more complete occupancy of the 17α pocket in the glucocorticoid receptor [12] and higher glucocorticoid receptor binding affinity than FP [13]. As part of the overall phase II development plan investigating FF, dose-ranging studies in asthma patients have demonstrated that FF has a favourable efficacy and safety profile when administered once-daily in the evening [14-16].

The aims of the current study were to compare the efficacy and safety of once-daily versus twice-daily FF regimens with each other and with placebo in patients uncontrolled on a non-corticosteroid controller or short-acting beta_2 _agonist (SABA) alone. The study specifically tested the hypothesis that a once-daily regimen is not inferior to a twice-daily regimen with respect to lung function (evening pre-dose forced expiratory volume in 1 second (FEV_1_)) after 4 weeks' treatment. FP once-daily and twice-daily regimens were included as active controls to confirm that the primary efficacy variable of trough FEV_1 _(measured pre-dose in the evening) on Day 28 was sensitive enough to detect differences between active treatments and placebo.

## Methods

### Study design

This was a randomized, placebo-controlled crossover study designed to compare the efficacy and safety of 28 days' treatment of FF given as a once-daily and twice-daily regimen in adolescents and adults with asthma. The study was approved by local ethics review committees and was conducted in accordance with the Declaration of Helsinki and Good Clinical Practice guidelines at 16 investigative sites in the USA between October 2008 and March 2009. All patients gave written informed consent. The trial is registered as NCT00766090 on the Clinicaltrials.gov registry and the sponsors' study number is FFA112202.

### Patients

Patients with moderate persistent asthma, aged 12 years or more with a pre-bronchodilator FEV_1 _of 40-85% of predicted normal value and reversibility of FEV_1 _to inhaled salbutamol of at least 12% and at least 200 ml were eligible for inclusion [17]. Patients were taking SABA and had not taken ICS for ≥ 8 weeks, but could have taken LABAs, xanthines, cromones, or leukotriene modifiers provided they had been stopped at screening. Patients had to refrain from using oral, parenteral, and depot forms of corticosteroids in the 8 weeks before screening and anti-IgE therapy in the 12 weeks before screening. We excluded patients who had smoked in the year before the study, those with a smoking history of > 10 pack-years, and individuals with a respiratory infection, life-threatening asthma, or asthma exacerbations requiring either hospitalization in the 6 months before screening or oral corticosteroids in the 8 weeks before screening. Drug therapy was withheld for baseline spirometry, treatment with LABAs and leukotriene modifiers was ceased the day before assessment, and patients could not take salbutamol during the 6-hour period before the clinic visit.

### Randomization

Eligible patients entered a run-in period of at least 7 days during which safety evaluations were conducted including a 24-hour urine collection for determination of cortisol excretion (see below). Patients were randomly assigned to either an FF group or an FP group, in a 7:2 ratio, respectively. To be eligible to enter the treatment period patients were required, at the end of the run-in period to exhibit the following; (I) evening FEV_1 _between 40% and < 80% and at least one of a daily symptom score of ≥ 1, rescue medication use on 4 of the last 7 days or PEF variability of ≥ 20% on 4 of the last 7 days; (2) evening FEV_1 _between ≥ 80% and 85% and at least one of a daily symptom score of ≥ 1, rescue medication use on all of the last 7 days or PEF variability of ≥ 20% on all of the last 7 days. Additionally patients were required to have a 24-hour urine cortisol sample available at the end of the run-in period. In the FF group all patients received drug via the NDPI and in the FP group via the Diskus™; thus although patients and investigators were blinded to which treatment they were receiving within a group, they were not blinded to whether they were allocated to an FF or an FP group. The central randomization schedule was generated by the sponsor using a validated computerized system (RandAll). The Registration and Medication Ordering System (RAMOS), an automated, interactive telephone based system, was used by the investigator or designee to register and randomize the patient and receive medication assignment information. Treatment assignment could be unblinded only in an emergency, through a call to the interactive telephone system.

### Treatments

Patients were assigned to 1 of 12 possible treatment sequences (table 1), each sequence comprising three 28-day treatment periods separated by two 2-week washout periods. Six sequences contained FF 200 μg once daily in the evening (with placebo in the morning), FF 100 μg twice daily and matching placebo twice daily dosed from a novel dry powder inhaler. Six sequences contained FP 200 μg once daily in the evening (with placebo in the morning), FP 100 μg twice daily and matching placebo twice daily dosed from a Diskus™ inhaler. The difference between the delivery devices used to deliver FF and FP meant that investigators could distinguish whether patients were assigned to an FF or FP sequence, but were double-blind to whether placebo or either of the two active regimens were being administered.

**Table 1:** Distribution of patients between treatment sequences

Sequence	Allocation ratio	Treatments			Delivery device
			
		Period 1	Period 2	Period 3	
1	7	Placebo	FF 200 μg OD	FF 100 μg BD	Novel dry powder inhaler
2	7	Placebo	FF 100 μg BD	FF 200 μg OD	
3	7	FF 100 μg BD	Placebo	FF 200 μg OD	
4	7	FF 100 μg BD	FF 200 μg OD	Placebo	
5	7	FF 200 μg OD	Placebo	FF 100 μg BD	
6	7	FF 200 μg OD	FF 100 μg BD	Placebo	

7	2	Placebo	FP 200 μg OD	FP 100 μg BD	Diskus™ inhaler
8	2	Placebo	FP 100 μg BD	FP 200 μg OD	
9	2	FP 100 μg BD	Placebo	FP 200 μg OD	
10	2	FP 100 μg BD	FP 200 μg OD	Placebo	
11	2	FP 200 μg OD	Placebo	FP 100 μg BD	
12	2	FP 200 μg OD	FP 100 μg BD	Placebo	

FF = fluticasone furoate; FP = fluticasone propionate; BD = twice daily;

OD = once daily.

From the screening visit onwards, no additional asthma medications were allowed except for rescue salbutamol. Intranasal and topical corticosteroids, and oral, ocular, and intra-nasal antihistamines were permitted.

### Outcome measurements

The primary endpoint was the pre-dose, pre-rescue bronchodilator FEV_1 _on the evening of Day 28 of the treatment period. The protocol required that spirometry was performed on Days 0 and 28 at 8.00 pm +/- 3 hours, at least 6 hours after the last administration of salbutamol and within 1 hour of the time of the Day 0 measurement.

### Safety evaluation

Adverse events (AEs) were coded using Medical Dictionary for Regulatory Activities (MedDRA, version 11). Safety assessments included routine laboratory tests, vital signs and oropharyngeal examination, and change in 24-hour urinary cortisol (UC) excretion between study baseline and the end of each 28-day treatment period. Patients who had asthma exacerbations (defined as any worsening of asthma that required emergency intervention, hospitalization, or treatment with an asthma medication not allowed by the study protocol) were withdrawn from the study.

### Statistical analysis

The intent-to-treat (ITT) population comprised all patients who received at least one dose of study medication; the per-protocol (PP) population was the subset of patients in the ITT population who completed at least one treatment period without a protocol deviation. Both populations were used to assess the primary comparison of FF once daily versus FF twice daily. For the assessment of differences between active and placebo groups, the ITT population was used. The PP population was considered to be a supportive analysis. The UC population consisted of patients who had urine samples with no confounding factors that would limit the analysis of UC.

Assuming an average within-patient standard deviation in pre-bronchodilator evening trough FEV_1 _of 210 ml and a non-inferiority limit of -110 ml, 84 completed patients would be required in the FF patient set to demonstrate non-inferiority of FF 200 μg once daily relative to FF 100 μg twice daily with 92% statistical power and a 2.5% one-sided significance level. For the superiority comparisons with placebo, this number of patients would enable detection of a difference of 200 ml between each of the FF groups and placebo with > 99% power. For the FP patient set, the target number of completed patients (*n *= 24) would enable detection of a 200 ml difference between FP dosed once daily or twice daily and placebo with 91% power, based on a 2-sided 5% significance level and a within-subject standard deviation of 210 ml.

For the primary efficacy analysis, comparison of treatment differences was performed using mixed model analysis of covariance (ANCOVA) with fixed effects for treatment, period, sex, and age and including period baseline as part of a bivariate response. In this analysis, ANCOVA was also used to compare treatment differences in UC excretion, with treatment, period, age, sex, and study baseline as fixed effects and patient as a random effect. For each treatment group, least square (LS) mean values were calculated for absolute pre-dose FEV_1 _and change in pre-dose FEV_1 _from period baseline. All analyses were pre-planned before the study blind was broken. No subgroup analyses were performed.

## Results

### Study population

**Figure 1:**
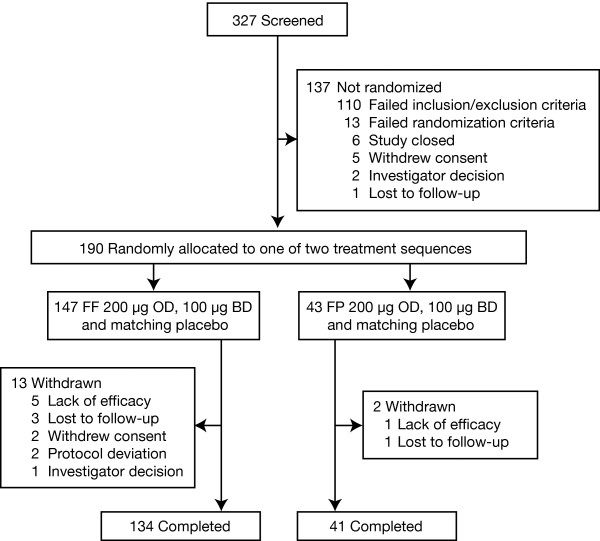
**Disposition of patients**. BD = twice daily; OD = twice daily.

Of 190 patients randomly assigned to study treatment, 147 were assigned to one of the six FF sequences and 43 to one of the six FP sequences; 134 and 41 patients, respectively, completed the study. Reasons for failure at the screening stage and reasons for withdrawal after the randomization stage are shown in Figure 1. The ITT population consists of all 190 patients who were randomized and 177 patients met the criteria to be included in the PP population. Baseline demographic and clinical characteristics of patients assigned to the two sets of treatment sequences (FF and FP) are shown in table 2. Asthma was generally long-standing with 164 patients (86%) having asthma for at least 5 years.

**Table 2:** Baseline characteristics of patients in each set of treatment sequences

	FF sequences (*n *= 147)	FP sequences (*n *= 43)	Total (*n *= 190)
Age (years)	31.4 (15.30)	35.2 (16.03)	32.3 (15.51)
Range	12-68	12-76	12-76
Females, n (%)	87 (59)	21 (49)	108 (57)
Race (%)			
White	90 (61)	22 (51)	112 (59)
African American/ African heritage	50 (34)	20 (47)	70 (37)
Other	7 (5)	1 (2)	8 (4)
History of atopy, n (%)	93 (63)	27 (63)	120 (63)
Lung function at screening			
Reversibility of FEV_1 _(%)	27.20 (13.667)	27.52 (16.449)	27.27 (14.298)
Reversibility of FEV_1 _(ml)	608.2 (304.64)	591.4 (367.47)	604.4 (318.98)
Lung function at study baseline			
Pre-bronchodilator FEV_1 _(L)	2.296 (0.6176)	2.293 (0.6990)	2.296 (0.6350)
Pre-bronchodilator FEV_1_ (% predicted)	69.85 (9.704)	67.73 (11.204)	69.37 (10.071)

Values are mean and standard deviation unless stated. Data shown are for the ITT population.

FEV_1 _= forced expiratory volume in 1 second; FF = fluticasone furoate;

FP = fluticasone propionate

### Efficacy

The mean values of pre-dose FEV_1 _on Day 28 in each treatment group and the mean changes compared with period baseline (Day 0) are shown in table 3 for both ITT and PP populations. Pre-dose FEV_1 _increased in all groups, but the mean increases in the four active treatment groups were approximately twice those in the placebo group. The differences versus placebo were statistically significant in all four active treatment groups, as assessed in the ITT population. For FF 200 μg once daily, FF 100 μg twice daily and FP 100 μg twice daily, the p value for the difference was < 0.001, while for FP 200 μg once daily the p value for the difference was 0.02.

**Table 3:** Evening pre-dose FEV_1 _on Day 28 of treatment and improvement from period baseline for each treatment regimen (all placebo treatments were pooled for these analyses)

	Placebo	FF 200 μg OD	FF 100 μg BD	FP 200 μg OD	FP 100 μg BD
**Number of patients**	187	140	142	42	43
**LS mean**, ml (SE)	2605 (43.4)	2714 (44.4)	2703 (44.3)	2693 (53.5)	2737 (53.3)
**LS mean change from period baseline**, ml (SE)	112 (18.6)	221 (20.9)	210 (20.7)	199 (36.5)	244 (36.1)
**LS mean difference** (active-placebo), ml (95% CI)	NA	108 (64-153); p < 0.001	98 (54-142); p < 0.001	87 (14-161); p = 0.020	132 (59-205); p < 0.001
**LS mean difference** (FF 200 μg OD-FF 100 μg BD), ml (95% CI)	NA	11 (-35-56); p = 0.641	NA	NA	NA

Absolute values and all differences are LS means, with 95% CI for non-inferiority and p values for superiority analysis for all comparisons between treatments. Data shown are for the ITT population.

BD = twice daily; CI = confidence interval; FF = fluticasone furoate;

FP = fluticasone propionate; LS = least square, NA = not applicable; OD = once daily; SE = standard error

**Figure 2:**
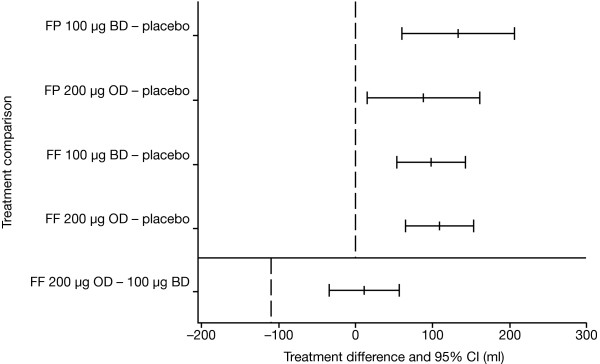
**Mean treatment difference (and 95% CI) adjusted for treatment, period, sex and age, for comparisons between active treatments and placebo and between the two FF dosage regimens (ITT population). Dotted line at 0 shows the point at which the two interventions would have an equal effect on pre-dose FEV_1_. The lower dotted line (for the FF 200 μg OD vs. FF 100 μg BD comparison) shows the predefined -110 ml threshold for non-inferiority of FF 200 μg OD versus FF 100 μg BD**. BD = twice daily; CI = confidence interval; OD = twice daily; FF = fluticasone furoate; FP = fluticasone propionate

In the ITT population, the lower 95% confidence interval (CI) for the mean difference between FF 200 μg once daily and FF 100 μg twice daily in pre-dose FEV_1 _on Day 28 was -35 ml (LS mean difference of 11 ml) (table 3; Figure 2). This difference was within the pre-defined limit of -110 ml, thus demonstrating non-inferiority of the FF 200 μg once-daily regimen. Similar results were obtained from the non-inferiority analysis in the PP population: the lower 95% CI for the treatment difference was -49 ml (LS mean difference 0 ml). Data from patients treated with FP indicated numerically reduced improvement in pre-dose FEV_1 _with the 200 μg once-daily dose in comparison with 100 μg twice daily, although no statistical comparison of these groups was performed.

### Safety

No serious AEs were reported and no AEs led to permanent discontinuation of drug or to patient withdrawal. The frequency of on-treatment AEs was higher in the FF 200 μg once-daily, FF 100 μg twice-daily and placebo NDPI groups (16%, 18%, and 14%, respectively) than in the FP 200 μg once-daily, FP 100 μg twice-daily and Diskus™ placebo groups (5%, 7% and 12% respectively). Upper respiratory tract infections (URTI) were the most commonly reported AEs, occurring in 5% of patients in each of the FF groups and 1% in the placebo group; no other AEs were reported by more than 1% of patients in either of the FF groups or the placebo group during the treatment period (table 4). However, only three of the AEs reported (headache/dry throat, FF 100 μg twice daily; tachycardia, FP 200 μg once daily) were considered to be potentially drug-related. One patient reported dysphonia (FP 200 μg once daily), but there were no cases of oral candidiasis.

**Table 4:** Number and proportion of patients reporting AEs during treatment, for events reported by at least 1% of patients in the FF or placebo arms

Number of patients reporting event, n (%)	Placebo nDPI (*n *= 145)	Placebo DISKUS (*n *= 42)	FF 200 μg OD (*n *= 140)	FF 100 μg BD (*n *= 142)	FP 200 μg OD (*n *= 42)	FP 100 μg BD (*n *= 43)
Any on-treatment AE	21 (14)	5 (12)	22 (16)	26 (18)	2 (5)	3 (7)
URTI	2 (1)	0	7 (5)	7 (5)	0	0
Sinusitis	0	1 (2)	1 (< 1)	2 (1)	0	0
Pharyngitis	2 (1)	0	1 (< 1)	0	0	0
Cellulitis	2 (1)	0	0	0	0	0
Tooth infection	0	0	2 (1)	0	0	0
Cough	0	0	0	2 (1)	0	0
Headache	2 (1)	0	2 (1)	0	0	0
Tension headache	0	0	2 (1)	0	0	0

Data shown are for the ITT population.

AE = adverse events; BD = twice daily; FF = fluticasone furoate; OD = once daily; URTI = upper respiratory tract infections.

Asthma exacerbations occurred in five (3%) patients on placebo, and one (< 1%) patient on FF 200 μg once daily. None of the exacerbations were severe enough to require hospitalization.

**Figure 3:**
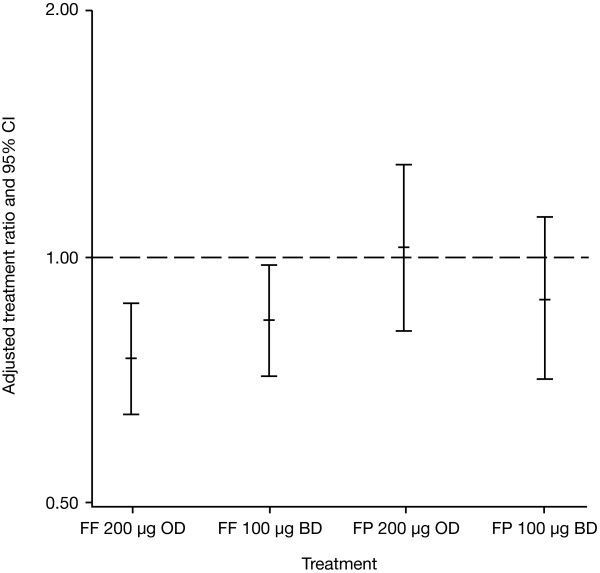
**Treatment differences for 24-hour urinary cortisol excretion on Day 28 of treatment, expressed as the adjusted ratio (active:placebo) of the absolute excretion values (UC population)**. BD = twice daily; CI = confidence interval; OD = twice daily; FF = fluticasone furoate; FP = fluticasone propionate

UC excretion data were analyzed in 170 patients with adequate 24-hour collections at study baseline and Day 28. The Day 28 ratio to placebo was statistically significantly lower in the FF 200 μg once-daily and FF 100 μg twice-daily arms (ratio 0.75, p < 0.001 and 0.84, p = 0.020, respectively), but the ratios with FP were not statistically significant for the FP 200 μg once-daily and FP 100 μg twice-daily arms (ratio 1.03, p = 0.808 and 0.89, p = 0.338, respectively) (Figure 3). No AEs associated with abnormal urinary-free cortisol were reported. There were no clinically important changes in any laboratory test parameter or vital signs with any study treatment during any of the treatment periods.

## Discussion

In this crossover study in adolescents and adults with moderate asthma, the same daily dose of a novel ICS, FF, administered once daily in the evening or as a twice-daily regimen was compared over a 28-day treatment period. For the primary efficacy variable of evening pre-dose FEV_1_, FF 200 μg once daily in the evening was non-inferior to FF 100 μg twice daily. All four active treatment arms were associated with significantly higher pre-dose FEV_1 _values than placebo. With FP, a numerically higher increase in pre-dose FEV_1 _with twice-daily dosing than with once-daily dosing was observed. The difference between once and twice daily FP was in line with differences previously reported for FP once versus twice daily [18,19], although this supports the current indication for twice-daily dosing of FP in asthma the study was not powered nor designed to assess differences between once-daily and twice-daily dosing of FP, only to assess differences between either FP dosing regimen and placebo. FF appears to be suitable for once-daily dosing as both once-daily and twice-daily dosing (same total daily dose) produced similar improvements in lung function,. The efficacy results for FF in the current study are consistent with the results of three dose-ranging studies in patients with different levels of asthma severity, in which 8 weeks of FF administered once daily in the evening produced superior improvements in lung function and symptoms relative to placebo at doses of 50-800 μg once daily [14-16].

Both regimens of FF were well tolerated in this study and AE reporting rates were similar to placebo, especially when considering the AEs reported for placebo and FF using the NDPI, and placebo and FP using the Diskus™. It is possibly the case that the higher incidence of AEs reported with the NDPI regardless of treatment (placebo or FF) resulted from a lack of familiarity with the device. There was only one asthma exacerbation among patients receiving FF and no dysphonia or oropharyngeal candidiasis. URTIs were the only event to be reported more often in the FF groups than with placebo, and were not associated with loss of asthma control. A reduction in UC of 16% and 25% (relative to placebo) was observed with FF 100 μg BD and FF 200 μg OD, respectively, and this finding contrasts those of other FF dose-ranging studies in which patients with asthma did not show any UC suppression relative to placebo after 8 weeks' treatment at doses up to 600 μg once daily [14-16]. There was a numerical reduction in UC of the same magnitude (19%) in the FP 100 μg twice-daily regimen. No adverse safety events were recorded in the current study that could be attributed to cortisol suppression. Further studies are needed to assess the magnitude of any potential effect of cortisol suppression in susceptible patients.

The crossover design used for the current study had the advantage of reducing potential variability compared with a parallel-group design. The study was not completely double-blind, as the differences in the inhaler devices used to deliver FF and FP enabled investigators to distinguish between those two groups. Patients may also have known whether they received FF or FP, but they had no involvement or choice in their treatment sequence or choice of active drug. However, patients received only one active treatment (FF or FP) throughout the study and no formal statistical comparisons were made between FF and FP. Given the considerations of trial practicality, we believe that the 2-week washout period between treatments was adequate for lung function and UC to return to baseline; this is consistent with the recommended minimum time reported for studies on ICS treatments [20]. The numbers of patients in the ITT and PP populations exceeded the numbers stipulated by the sample size calculation, as a higher than expected proportion of screened patients met the eligibility criteria for study treatment. We do not believe the use of different devices for FF and FP should be considered as a confounder for the main study outcomes, although it could be considered as a limitation of the study. Furthermore, trough FEV_1 _was the sole efficacy endpoint of this study and as such the non-inferiority of FF 200 μg once-daily dosing to FF100 μg twice-daily dosing cannot be inferred for other measures of treatment responsiveness such as PEFR, symptoms and exacerbations.

A once-daily ICS regimen has the potential to improve adherence by offering greater convenience while ensuring continuous 24-hour control of inflammation and symptoms. Lack of adherence to ICS treatment in asthma patients is a predictor of suboptimal disease control and poor outcome in children and adults [8,10,21]. A retrospective study in children and adults showed that in patients who needed asthma-related emergency care, persistence with ICS use in the previous 12 months was low (fewer than three prescriptions filled), and that despite an increase in the number of prescriptions dispensed in the month of the emergency event, dispensing rate returned to the level observed previously in the second month after the event [22]. In another retrospective analysis, adolescents/young adults with mild asthma receiving mometasone furoate once daily showed better adherence and asthma control than those receiving other twice-daily ICS treatments [23]. However, while a once-daily regimen is approved for some agents for maintenance treatment of mild asthma [6], the recommended dosing frequency for the majority of ICS and for most patients is twice daily.

The current study used a once-daily evening dose regimen. Previous studies have compared evening with morning dosing for once-daily ICS regimens. In a previous study on FF, a 400 μg once-daily evening dose regimen had similar efficacy to a 200 μg twice-daily regimen, but the 400 μg once-daily morning dose, although effective, was less effective than FF 200 μg twice daily [24]. Data on other ICS also suggest improved efficacy for evening dosing. Triamcinolone once daily was more effective when the dose was given in the afternoon than in the morning [25]. Ciclesonide 200 μg once daily had a significantly greater improvement from baseline in morning PEFR with evening compared with morning dosing [26]. Mometasone furoate 200 μg taken in the evening appeared superior to morning dosing as measured by change in FEV_1_, forced vital capacity, and PEFR from baseline after 12 weeks (although formal statistics were not applied) [27]. In contrast, there appeared to be no difference between morning and evening once-daily dosing for budesonide compared with twice-daily dosing [28].

## Conclusions

In conclusion, four weeks' treatment with FF given as a 200 μg dose once daily in the evening has superior efficacy and similar tolerability compared with placebo in patients with moderate asthma, and is non-inferior to a FF 100 μg twice-daily regimen as measured by pre-dose FEV_1 _response. Some cortisol suppression was noted with FF, although this was not observed in previous studies that used higher doses of FF and for longer treatment durations. Although confirmatory studies are required, the data support the use of FF as a once-daily, evening dosed, treatment in asthma.

## List of abbreviations

AE: Adverse event; ANCOVA: Analysis of covariance; CI: Confidence interval; FEV_1_: Forced expiratory volume in 1 second; FF: Fluticasone furoate; FP: Fluticasone propionate; ICS: Inhaled corticosteroid; ITT: Intent-to-treat; LABA: Long-acting beta_2 _agonist; LS: Least square; NDPI: Novel Dry Powder Inhaler; PEFR: Peak expiratory flow rate; PP: Per protocol; UC: Urinary cortisol; URTI: Upper respiratory tract infection; SABA: Short-acting beta_2 _agonist

## Competing interests

AW has served as consultant to Almirall, AstraZeneca, Chiesi, GlaxoSmithKline, Merck Sharpe and Dohme and Novartis; has received lecture fees and travel expenses for attendance at ATS and ERS meetings from GlaxoSmithKline; has been PI on clinical trials conducted by University Hospital of South Manchester. ERB has served as a consultant to and received lecture fees from GlaxoSmithKline; and has performed clinical trials for GlaxoSmithKline, which have been administered by his employer Wake Forest University Health Sciences. EDB has served as a consultant to and received lecture fees from GlaxoSmithKline; and his institution has received remuneration for participation in clinical trials sponsored by GlaxoSmithKline. WWB has served as a consultant to AstraZeneca, Boehringer Ingelheim, Novartis and TEVA; served on advisory boards for Altair, Amgen, Centocor, GlaxoSmithKline, Johnson & Johnson, Merck Sharpe and Dohme, Pfizer and Wyeth; received lecture fees from Merck Sharpe and Dohme; and received research funding from AstraZeneca, Ception, GlaxoSmithKline, MedImmune and Novartis. JL has served as a consultant to and received lecture fees from AstraZeneca, GlaxoSmithKline, Merck Sharpe and Dohme, Novartis and UCB Pharma; has been partly covered by some of these companies to attend previous scientific meetings including the ERS and the AAAAI; and has participated in clinical research studies sponsored by AstraZeneca, GlaxoSmithKline, Merck Sharpe and Dohme, and Novartis. JL is also editor of Respiratory Research and recused himself fully from the editorial process of this manuscript. NGS, LF, LJ and BH are employees of and hold stock in GlaxoSmithKline.

## Authors' contributions

All authors developed the design and concept of the study. NGS, LF, LJ, and BH approved the statistical plan. NGS served as the clinical investigation lead and in that role coordinated generation of the protocol and data gathering. LF led the statistical analysis. All authors vouch for the accuracy and completeness of the data and the data analysis. All authors read and approved the final manuscript.
